# Topoisomerase IIα-mediated stemness response in reactive astrocytes to traumatic brain injury

**DOI:** 10.7150/thno.111923

**Published:** 2025-04-13

**Authors:** Shangyao Qin, Xiao Huang, Yimin Yuan, Hong Liu, Jiali Li, Ziwei Dai, Zhida Lan, Yingyan Pu, Cheng He, Zhida Su

**Affiliations:** 1 Institute of Neuroscience and Key Laboratory of Molecular Neurobiology of Ministry of Education, Naval Medical University, Shanghai 200433, China.; 2 Department of Anesthesiology, Sichuan Academy of Medical Sciences and Sichuan Provincial People's Hospital, University of Electronic Science and Technology of China, Chengdu, 610072, China.; 3 Department of Pain Medicine, School of Anesthesiology, Naval Medical University, Shanghai 200433, China.; 4 Department of Anatomy, College of Basic Medicine, Naval Medical University, Shanghai 200433, China.

**Keywords:** TOP2a, reactive astrocytes, stem cell properties, traumatic brain injury

## Abstract

**Rationale:** As a highly plastic population, parenchymal astrocytes have demonstrated the capacity to become activated and recapitulate neurogenic potential in response to traumatic central nervous system (CNS) injuries, representing a latent reservoir for neuronal regeneration in non-neurogenic brain regions. However, the extrinsic and intrinsic factors regulating this process remain poorly characterized. Elucidating these molecular mechanisms is crucial to harnessing the regenerative potential of reactive astrocytes in CNS repair.

**Methods:** A multidisciplinary approach combining immunostaining, western blotting, RNA interference (RNAi), gene knock out and fate-mapping was used to investigate the role of topoisomerase IIα (TOP2a) in regulation of the stemness response in reactive astrocytes to traumatic brain injury (TBI).

**Results:** Both *in vitro* and *in vivo* analyses demonstrated that TBI induces a stem cell-like response in reactive astrocytes concomitant with TOP2a upregulation. Pharmacological inhibition or genetic deletion of TOP2a significantly attenuated this stemness response. Neurosphere culture assay indicates that TOP2a might act as a downstream factor of Sonic Hedgehog (SHH) signaling to mediate the acquisition of stem cell potential.

**Conclusions:** This study identifies TOP2a as a pivotal intrinsic regulator of astrocytic stem cell potential in the injured brain, which will advance our understanding of the molecular underpinnings of the stem cell response and its therapeutic application in neural regeneration.

## Introduction

CNS injury and disease frequently cause irreversible loss of neurons, resulting in persistent neurological disability. Neural stem cells (NSCs) are multipotent cells that possess the capacity for self-renewal and differentiation into neurons, astrocytes, and oligodendrocytes 1. NSCs in the adult mammalian CNS have been proposed as an important resource for endogenous neural regeneration via proper activation 1. However, the adult NSCs are mostly confined to the germinal niches of the subventricular zone (SVZ) and hippocampal subgranular zone (SGZ), which limits their application for CNS repair. It is important to identify a cell of origin (if exist) for NSCs outside the neurogenic niches in the adult mammalian CNS.

As the most abundant neural cells, astrocytes are broadly distributed throughout the CNS. It has been widely accepted that astrocytes are a cell population with high heterogeneity and retain a high level of plasticity 2-4. Surprisingly, adult NSCs in SVZ and SGZ are identified as astrocyte-like cells that exhibit structural and biological signatures of astrocytes 5-8. By overexpression of specific transcription factors (TFs), endogenous parenchymal astrocytes can be reprogrammed into neurons 9-13. Crucial to attract researchers' interest are the demonstrations that ubiquitous astrocytes become activated and recapitulate stem cell potential in response to diverse types of invasive CNS injury, such as stab wounding and cerebral ischemia 14-16. Following brain injury, astrocytes are capable of re-entering the cell cycle in the process of reactive astrogliosis and acquire an NSC character, suggestive of a potential of parenchymal astrocytes to function as dormant NSCs 14, 15, 17, 18. These cells might provide an alternative new resource for endogenous repair in the case of local or widespread neuronal damage. However, little is known about how reactive astrocytes acquire or reactivate the NSC potential.

Topoisomerases are nuclear enzymes that resolve topological problems associated with DNA during various genetic activities such as replication, transcription, recombination, repair, and chromatin remodeling 19. They regulate the overwinding or underwinding of DNA helix by providing a transient DNA break. Based on the catalytic function, reaction mechanism, amino acid sequence and structure, topoisomerases are generally classified into type I (TOP1) and II (TOP2) 19, 20. TOP1 introduces single DNA strand break whereas TOP2 cleaves both stands. In mammalian cells, TOP2 includes α and β isoforms; they have identical catalytic activities but distinct localization patterns during mitosis. TOP2a is involved in the cellular proliferation and pluripotency, while TOP2β plays key roles in cell differentiation and neurogenesis 21-23. Although it has well been documented that topoisomerases resolve torsional stress, their functions in gene regulation, especially during cellular differentiation, remain elusive 24.

In the present study, we showed that in response to stab wound-induced traumatic brain injury (TBI), a subpopulation of reactive astrocytes took on stem cell-related properties, such as proliferating, expressing NSC-associated proteins and forming neurospheres when cultured *in vitro*. Interestingly, TOP2a was found to express in reactive astrocytes but not quiescent astrocytes. Through pharmacological inhibition, RNA interference and conditional knock-out mouse studies, we indicated that TOP2a was necessary to elicit the stem cell response of reactive astrocytes *in vitro* and *in vivo*. These findings suggested TOP2a as a key intrinsic regulator of the cellular plasticity of reactive glia.

## Materials and Methods

### Animals

C57BL/6 mice were purchased from Shanghai Ling Chang Biotech Co., Ltd. All transgenic mice were obtained from the Jackson Laboratory except where specifically noted. We generated Top2a^f/f^ mice and maintained them on C57BL/6 background 24. For genetic tracing of astrocytes, Gfap-Cre mice were crossed with Rosa-GFP to generate Gfap-Cre;Rosa-GFP transgenic mice. For selective deletion of TOP2a in astrocytes, Top2a^f/f^ mice were crossed with Gfap-CreER^T2^ mice to generate Gfap-CreER^T2^;Top2a^f/f^ transgenic mice. For genetic tracing of astrocytes and selective deletion of TOP2a in astrocytes, Aldh1l1-CreER^T2^ transgenic mice (Provided by Professor Tian-Ming Gao, Southern Medical University) 25 and a fluorescent reporter line (Ai14, Rosa-tdTomato) were crossed with Top2a^f/f^ mice to generate Aldh1l1-CreER^T2^; Rosa-tdTomato; Top2a^f/f^. Tamoxifen (Sigma-Aldrich, Cat#T5648) was administered to induce recombination in the CreER^T2^/loxP system as previously described 24. Littermates that did not harbor any transgene were used as controls. Both adult male and female mice at 2-3 months of age were used unless otherwise stated. Animals were housed in plastic cages with disposable bedding on a normal 12 h light/dark cycle with food and water ad libitum. Experiments involving animals followed a protocol approved by the Animal Experimentation Ethics Committee of Naval Medical University (NMUMREC-2021-033) in strict accordance with NIH guidelines.

### TBI model

As a model of TBI with pronounced reactive gliosis 18, 26, stab wound injury was selected in our study. C57BL/6 or Gfap-CreER^T2^;Top2a^f/f^ mice at 2~3 months of age were selected to prepare the TBI model as previously described 27. After anesthetizing with 2% pentobarbital (30 mg/kg), the experimental animals were fixed on the operating table. The skull was drilled and a medical scalpel was used to perform a unilateral stab wound in the right somatosensory cortex (Bregma from 1 mm to -0.1 mm, anterior/posterior 1.7 mm, 1.1 mm deep). After surgery, animals were returned to their home cages and injected with buprenorphine (0.1 mg/kg) for postoperative pain control. The cerebral cortex contralateral to the stab wound was used as an uninjured control.

### Tamoxifen and BrdU Treatments

Tamoxifen (TAM) was dissolved in ethanol at a concentration of 10 mg/mL. To induce Cre activity *in vivo*, Gfap-CreER^T2^;Top2a^f/f^ mice were intraperitoneally injected with TAM at a daily dose of 10 μl/g body weight for 7 days, followed by a 7-day clearing period without TAM. To induce Cre activity in culture, cells were isolated from stab-wounded cerebral cortex of Aldh1l1-CreER^T2^;Top2a^f/f^;Rosa-tdTomato mice and treated with TAM (1 µM). For BrdU incorporation assay, mice were intraperitoneally injected with BrdU (100 mg/kg) once a day from the first day to the fifth day after injury.

### Immunohistochemistry

For immunohistochemistry, mice were deeply anesthetized with 2% pentobarbital (30 mg/kg) and subjected to transcardial perfusion with 4% paraformaldehyde. Brains were surgically dissected, post-fixed overnight and followed by cryoprotection with 30% sucrose at 4 °C for 48 h. Every fifth section was collected (around 10-15 sections per mouse) on a Leica cryostat set at 20-μm thickness. For immunocytochemistry, cells were fixed with 4% paraformaldehyde for 20 min at room temperature. Tissue sections or fixed cells were firstly permeabilized and blocked with 0.2% Triton X-100 and 3% BSA in 1✕PBS for 1 h, then they were overnight incubated at 4 °C with the primary antibodies (Table S1). Corresponding secondary antibodies Alexa Fluor 488, 555 or 647 (Jackson ImmunoResearch, 1:200) were used for indirect fluorescence. The nuclei were counterstained with Hoechst 33342 (Hst; Sigma-Aldrich, 1:1000). Images were acquired by Nikon E600FN microscope, Zeiss LSM510 and Leica confocal microscopy.

For BrdU staining, hydrochloric acid was used to denature DNA at the concentration of 2 mol/mL for 30 min at 37 °C. To neutralize excess hydrochloric acid, the brain sections were incubated with sodium tetraborate at the concentration of 0.1 mol/L at room temperature for 10 min. BrdU incorporation was detected with an anti-BrdU antibody by further fluorescent staining.

### Cell quantification *in vivo*

As previously described 24, 28, standard histological analysis protocols were used to quantify cells *in vivo*. In each condition, 3-5 animals were used and every fifth coronal section (25 µm) was collected from injured and uninjured brain. Confocal laser scanning (Zeiss LSM510; Leica) or epifluorescence (Nikon E600FN) microscopes were used to quantify immunopositive cells in sections. The total number of immunolabeled cells in each histological cross-section was counted at ×20 magnification by analyzing composite mosaics generated from 5-10 overlapping confocal images or digital scans acquired using a Nikon slide scanner system. A threshold was applied to the processed images to trace the contour of the coronal section and a grid of 50 µm × 50 µm positioned over the brain section using ImageJ to perform regional analysis. The quantification of immunostainings was based on analysis of at least five sections per animal and the results were expressed as number of cells per area. All data collection and analyses were performed by investigators blind to the treatment conditions.

### Western blot

The same tissue volume (about 0.2 cm^3^) prepared from the lesioned area of cerebral cortex or contralateral intact cortex of stab-wound mice was used for western blot analysis. Brain tissue was dissected and lysed in RIPA lysis buffer. The sample was centrifuged for 15 min at 4 °C before the lysate supernatant was collected. The concentration of protein was measured by a BCA assay (Beyotime Institute of Biotechnology, P0010). After that, the supernatant was mixed with sample buffer at 100 °C water bath for 10 min. The extract was separated by electrophoresis in 10% SDS-PAGE and transferred onto nitrocellulose membrane. The membrane was then blocked with 10% milk for 2 h and incubated with primary antibodies (Table S2) at 4 °C overnight. After incubation with corresponding HRP-conjugated secondary antibody for 1 h at room temperature, immunoreactive bands were visualized by using chemiluminescence reagents (ECL, Amersham) according to the manufacturer's instructions. The membranes were scanned with the Odyssey infrared imaging system and analyzed with Image Lab 2.0.

### Neurosphere culture

The culture of neurosphere was performed as previously described 27. After removing the skull in a sterile environment, a volume of tissue about 0.2 cm^3^ from the lesioned areas of stabbed cerebral cortex was isolated. The same tissue volume from corresponding anatomical areas of contralateral cortex was used as a control. Tissues were digested with trypsin at 37 °C for 25 min, and were dispersed into a single cell level. Finally, cells were seeded into 24-well plates at a density of 1-5 cells/μL and cultured with NSC medium containing FGF2 and EGF (both at 20 ng/mL, Invitrogen). After primary neurospheres were formed, by mechanical disruption, they were serially passaged under the same culture condition. Cultured neurospheres were treated with shRNA or chemical agents including ICRF-193 (Sigma-Aldrich), VP-16 (Sigma-Aldrich), PluriSIns#2 (also known as 1-Phenylcarbamoyl-5-fluorouracil, Fuchun Biotech), Cyclopamine (MedChemExpress) and SAG (Sigma-Aldrich). Their self-renewal ability was assessed by quantitative analysis of the number and size of neurospheres. For differentiation, secondary neurospheres were dissociated into single cells and seeded onto Matrigel-coated glass coverslips. Cells were further cultured for 6-10 days in the NSC medium without FGF2 and EGF.

### Plasmids construction and virus preparation

Lentiviral vectors encoding Top2a shRNA were constructed as described previously 11, in which gene expression was under the control of a modified human GFAP promoter. The sequences for shRNA are listed below: shRNA1 (5′-CCGGTGCCAAGAGCTTTGGATCAATTCAAGAGATTGATCCAAAGCTCTTGGCTTTTTTG-3′ and 5′-AATTCAAAAAAGCCAAGAGCTTTGGATCAATCTCTTGAATTGATCCAAAGCTCTTGGCA-3′), shRNA2 (5′-CCGGTGCGAGAAGCCTCTCATAAATTCAAGAGATTTATGAGAGGCTTCTCGCTTTTTTG-3′ and 5′-AATTCAAAAAAGCGAGAAGCCTCTCATAAATCTCTTGAATTTATGAGAGGCTTCTCGCA-3′) and shRNA3 (5′-CCGGTGCAGACTACATTGCCGTTTTTCAAGAGAAAACGGCAATGTAGTCTGCTTTTTTG-3′ and 5′-AATTCAAAAAAGCAGACTACATTGCCGTTTTCTCTTGAAAAACGGCAATGTAGTCTGCA-3′). The third generation, replication-deficient lentivirus was produced in HEK293T cells by transient transfections with lentiviral vectors and the packaging plasmids (pMDL, VSV-G and pREV). The virus-containing culture supernatants were collected, precipitated with polyethylene glycol 8,000 and concentrated by centrifugation. The knock-down efficiency of Top2a shRNA was determined by western blot. The nonsilenced group was used as a control.

### RNA-sequencing and bioinformatics analysis

Cultured neurospheres formed by reactive astrocytes isolated from stab-wounded cerebral cortex were harvested for RNA-sequencing (RNA-Seq) analysis three days after PluriSIns#2 or DMSO treatment. Following extraction of the total RNA from cultured neurospheres with Trizol reagent (Invitrogen), RNA-Seq was carried out on an Illumina platform. FastQC software (version 0.11.9, available at http://www.bioinformatics.babraham.ac.uk/projects/fastqc/) was used to analyze the data produced from the sequencer, and the reads were aligned to the mouse genome (mm10) using the software STAR (Spliced Transcripts Alignment to a Reference, version 2.6.1a) 29. In addition, the program Htseq-count (version 0.12.4) was used to count the alignment reads, and the the count matrix was normalized with DESeq2 R package (version 4.1.0). The differentially expressed genes (DEGs) were then identified using a screening criterion of log_2_ fold change (|log2FC|) *>* 1 and false discovery rate (FDR) *<* 0.05. The clusterProfiler R package was also used to annotate DEGs, which included Gene Ontology (GO) functional analysis and Kyoto Encyclopedia of Genes and Genomes (KEGG) metabolic pathway analysis.

### Quantitative RT-PCR

Neurospheres cultured *in vitro* were collected and lysed in 500 ul of TRIzol reagent. RNA product was reverse transcribed into cDNA using the RevertAid First Strand cDNA Synthesis Kit (Thermo Scientific Fermentas) following the manufacturer's protocol, and qPCR was performed with a LightCycler96 (Roche). Using the 2^- ΔΔCt^ method, the levels of Top2a gene transcripts were calculated and quantified after normalization to the expression of β-actin. Three independent experiments were performed with the relative-quantitative method. The primer sequences used in this study were as follows: Top2a (5'-GTAATTTTGATGTCCCTCCACGA-3' and 5'-TCAAGGTCTGACACGACACTT-3') and β-actin (5'-CTCGTCATACTCCTGCTTGC-3' and 5'-GAAGTGTGACGTGGACATCC-3').

### Statistical analysis

The statistical analysis was performed using GraphPad Prism software. All data were validated by at least three animals or experimental culture batches. The quantitative data were expressed as mean ± SEM. Student's *t*-test and one-way analysis of variance (ANOVA) with Tukey's post-hoc test were used to compare independent samples. P < 0.05 was used as a standard for considering statistically significant.

## Results

### TBI triggers stemness response of reactive astrocytes

**Figure 1:**
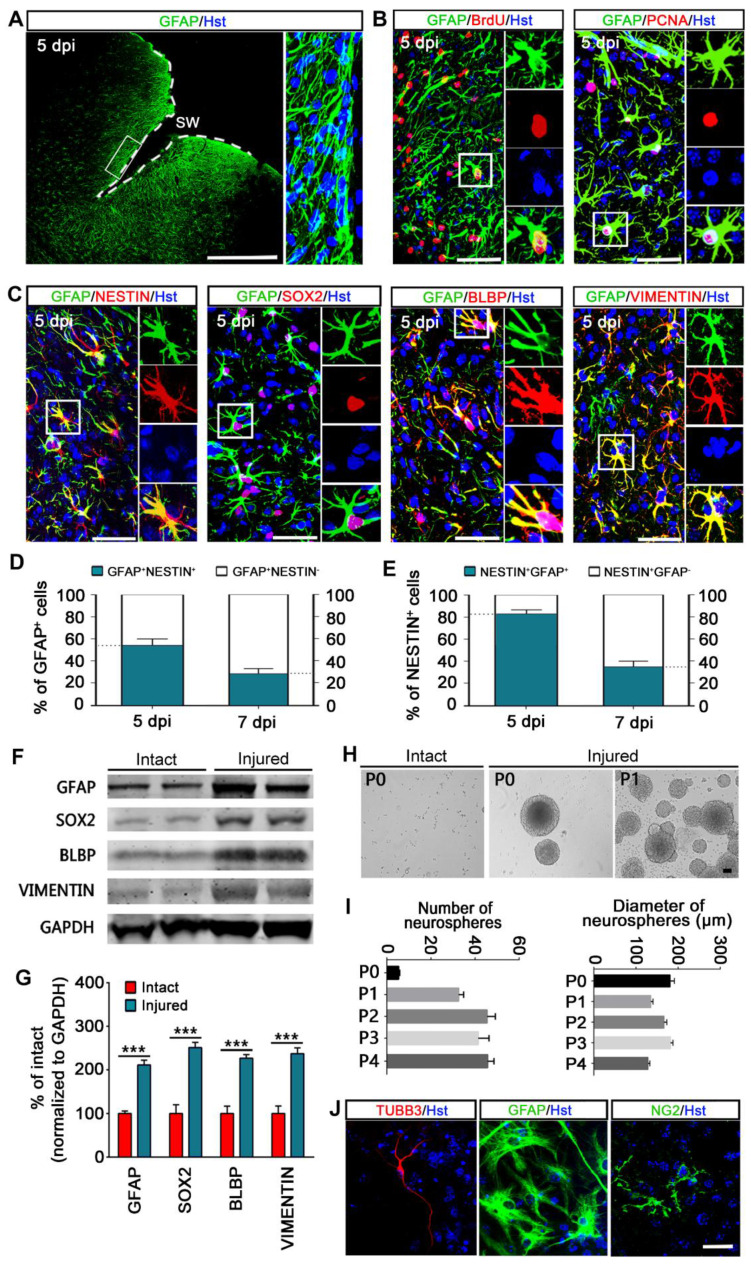
** Astrocytes are activated and acquire stem cell properties following stab wounding. (A)** Representative images showed the reactive astrocytes located in close vicinity to the lesion area. Higher magnifications (in right panel) showed that the activated astrocytes became hypertrophic and intensely expressed GFAP. **(B)** Reactive gliosis and proliferation of astrocytes in the injured cortex. **(C)** Immunostaining showed that reactive astrocytes expressed stem cell markers, NESTIN, SOX2, BLBP, and VIMENTIN. **(D and E)** Percentage and phenotype analysis of stab wound-induced reactive astrocytes by staining with GFAP and NESTIN at 5 and 7 dpi (n = 4). **(F and G)** Western blot analysis of the expression of intermediate filament protein GFAP and the stem cell markers, SOX2, BLBP, and VIMENTIN in the cortical region contralateral (intact) or ipsilateral (injured) to a stab wound, isolated at 5 dpi. The histogram in (H) depicts the quantitative analysis of western blot by densitometry. ***P < 0.001 by Student's *t*-test. **(H and I)** Neurospheres formed by cells isolated from the ipsilateral (injured) but not contralateral (intact) cerebral cortex 5 days after stab wounding. Representative micrographs of primary (P0) and secondary (P1) neurospheres were shown in (H). The number of each generation of neurospheres per 10,000 cells isolated was quantified after 12 days in culture, and the diameter of these neurosphers was also detected (I) (n = 5 mice). **(J)** Multiple lineage differentiation of primary neurospheres. Immunocytochemistry with the cell-type-specific markers was performed after differentiated for 7 days. White dashed line indicates the injured area. SW, stab wound. Scale bars: (A), 200 µm; (B), (C), (H) and (J), 50 µm.

To investigate astroglial response to invasive injury, we induced a stab wound in the right cerebral cortex of adult mouse (Figure S1A). Five days post injury (dpi), animals were euthanized and brains were prepared for *in vivo* and *in vitro* analysis. Compared with the cortex contralateral to the lesion site (intact cortex), stab wound resulted in massive activation of astrocytes adjacent to the injured area (Figure S1B and S1C, Figure 1A). The activated astrocytes showed intense GFAP immunoreactivity and became hypertrophic (Figure 1A, Figure S1C).

Normally, mature astrocytes are quiescent and largely stop proliferation, while BrdU incorporation assay indicated that GFAP^+^ reactive astrocytes showed a robust proliferative response over the 5 days following stab wounding and still proliferating at 5 dpi as measured by PCNA immunostaining (Figure 1B). The stab wound-induced proliferation ultimately resulted in significant astroglial hyperplasia in the region around the lesion site (Figure S1D).

Of note, many reactive astrocytes located in close vicinity to the lesion area were also found to express proteins characteristic of immature glia or NSCs, including NESTIN, SRY-box containing gene 2 (SOX2), brain lipid binding protein (BLBP) and VIMENTIN (Figure 1C). We did not observe NESTIN, BLBP or VIMENTIN expression on the contralateral side of the brain after stab wounding. As reported, SOX2 was expressed in both quiescent and reactive astrocytes, whereas we found that the number of SOX-positive cells was significantly increased on the ipsilateral side of the brain compared with the contralateral side of the brain (data not shown). Quantitative analysis revealed that about 54% and 28% of GFAP-positive reactive astrocytes were immunoreactive for NESTIN at 5 and 7 dpi, respectively (Figure 1D). Of the NESTIN-expressing cells, around 83% and 35% were GFAP positive at 5 and 7 dpi, respectively (Figure 1E). Importantly, the upregulated expression of GFAP, SOX2, BLBP and VIMENTIN in reactive astrocytes was further confirmed by western blot via isolating a defined volume of ipsilateral injured cortex and contralateral intact cortex of stab-wound mice (Figure 1F and 1G). Together, these findings suggest that astrocytes are activated in the stab wound-injured cortex, which inducing re-expression of markers of stemness in a subpopulation of reactive astrocytes *in vivo*.

Next, the stem cell response of stab wound-induced reactive astrocytes was further investigated in term of the potential for neurosphere formation *in vitro*. Five days following stab wounding, we isolated cells from peri-injury cortex in the injury side (injured) and cortical cells from the contralateral side of the brain were harvested as a negative control (intact). Cells were cultured in NSC growth medium with EGF and bFGF. After 12 days of culture, we observed that neural spheres were formed by cells isolated from injured but not intact cerebral cortex (Figure 1H). These injured cortex-derived neurospheres were capable of self-renewal and could be passaged on for at least five generations (Figure 1I). Of note, the number of primary neurospheres was significantly less than that of passaged neurospheres, whereas there was no significant difference in their diameter (Figure 1I). Importantly, these neurospheres could generate TUBB3^+^ neurons, GFAP^+^ astrocytes, and NG2^+^ oligodendrocyte lineage cells upon exposed to differentiation conditions for 7 days in culture, suggesting that they were multipotent (Figure 1J). Consistent with previous studies 15, 27, our data indicate that a subpopulation of stab wound-derived cortical cells activate the capacity to self-renew and generate all three CNS cell types (neurons, astrocytes and oligodendrocytes) in culture, suggestive of a stem cell response after TBI.

### Fate mapping analysis of reactive astrocytes' stem cell response to TBI

**Figure 2:**
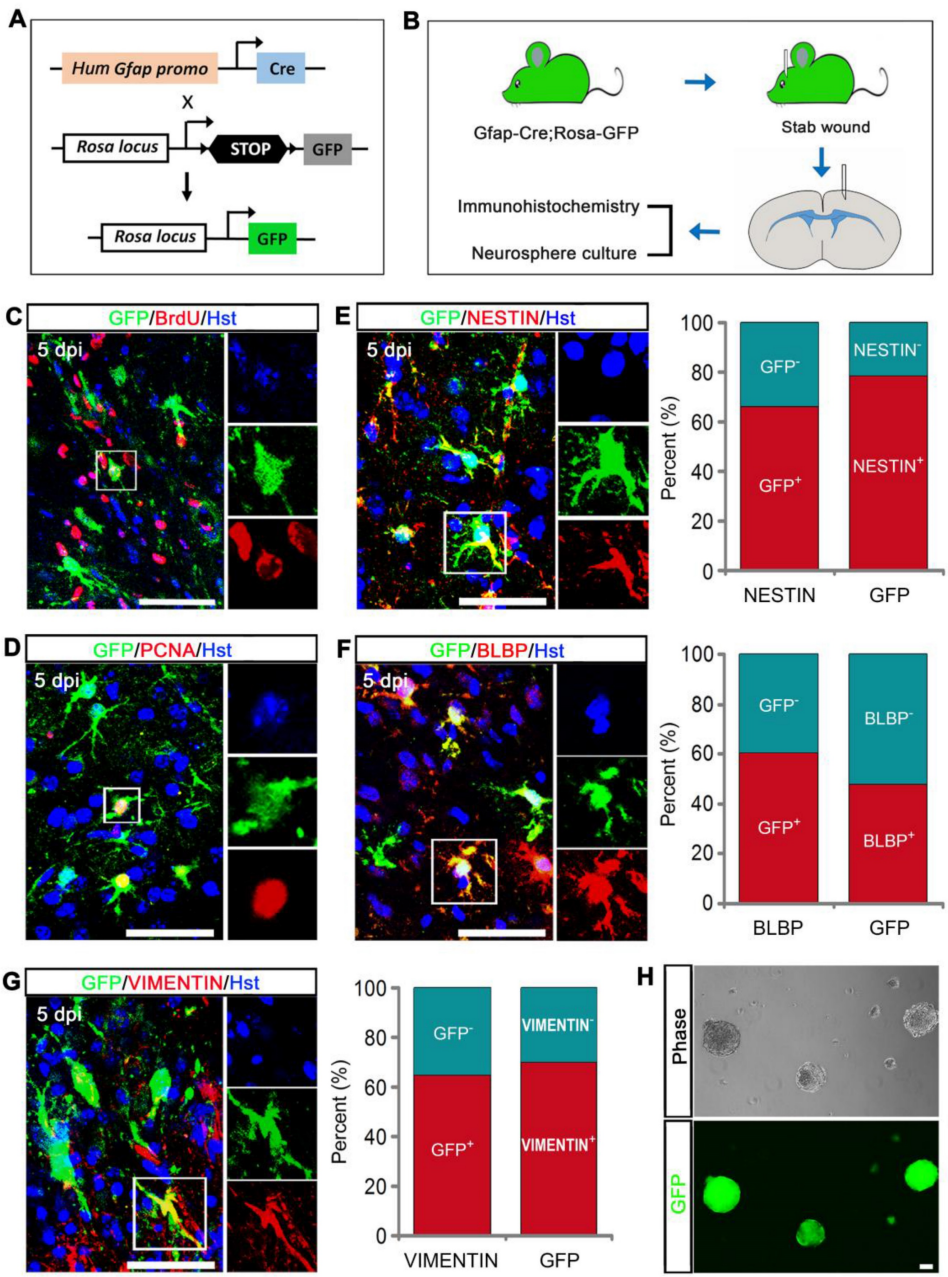
** Stem cell properties were analyzed using Gfap-Cre;Rosa-GFP transgenic mice. (A)** The genetic strategy to trace reactive astrocytes. **(B)** Schematic diagrams of experimental procedures. **(C and D)** Reactive gliosis and proliferation of GFP-traced astrocytes in the injured cortex. **(E-G)** Immunostaining showed that GFP-traced reactive astrocytes expressed stem cell markers, NESTIN, BLBP, and VIMENTIN. Quantitative analysis was also performed (n = 3). **(H)** Representative micrographs of neurospheres formed by cells isolated from the lesioned cerebral cortex of Gfap-Cre;Rosa-GFP transgenic mice 5 days after stab wound. Scale bars: 50 µm.

To further confirm that GFAP-positive reactive astrocytes contributed to the TBI-induced stem cell response, we generated Gfap-Cre;Rosa-GFP transgenic mice in which GFP was exclusively expressed in GFAP-positive cells (Figure 2A). After stab wound injury was induced on Gfap::Cre;Rosa::GFP mice, the animals were applied for immunohitochemical analysis and neurosphere culture (Figure 2B). Mice received the DNA base analog BrdU by daily intraperitoneal injection for consecutive 5 days starting immediately after stab wounding. Upon TBI, GFP-labeled cortical astrocytes adjacent to injury site became hypertrophic and incorporated BrdU (Figure 2C). The proliferative response of GFP-traced reactive astrocytes was also reflected by expression of the cell proliferation marker PCNA (Figure 2D). To further analyze the stem cell response to injury, we performed double immunostaining with GFP reporter and NSC-related markers, including NESTIN, BLBP, and VIMENTIN. Five days after stab wounding, 66.38% of NESTIN-positive cells were co-labeled by GFP, and 78.57% of GFP-labeled cells expressed NESTIN around the lesion site (Figure 2E). For BLBP, 60.71% of BLBP-positive cells were traced by GFP, and 47.72% of GFP-traced cells were immunoreactive for BLBP in the peri-injury area (Figure 2F). We also observed that 64.76% of VIMENTIN^+^ cells were co-stained with GFP, and 70.10% of GFP-labeled cell were VIMENTIN positive adjacent to injury site (Figure 2G). In addition, we carefully isolated cells from the lesioned areas of stabbed cerebral cortex that were far away from neurogenic niches and cultured them in NSC growth medium with EFG and bFGF. Twelve days later, we observed the formation of neurosphere, the majority of which were GFP-positive (Figure 2H). Altogether, these findings indicate that the cells acquiring stem cell properties following stab wound are mainly derived from injury-induced reactive astrocytes.

### TOP2a is elicited to express in reactive astrocytes in the cerebral cortex after TBI

**Figure 3:**
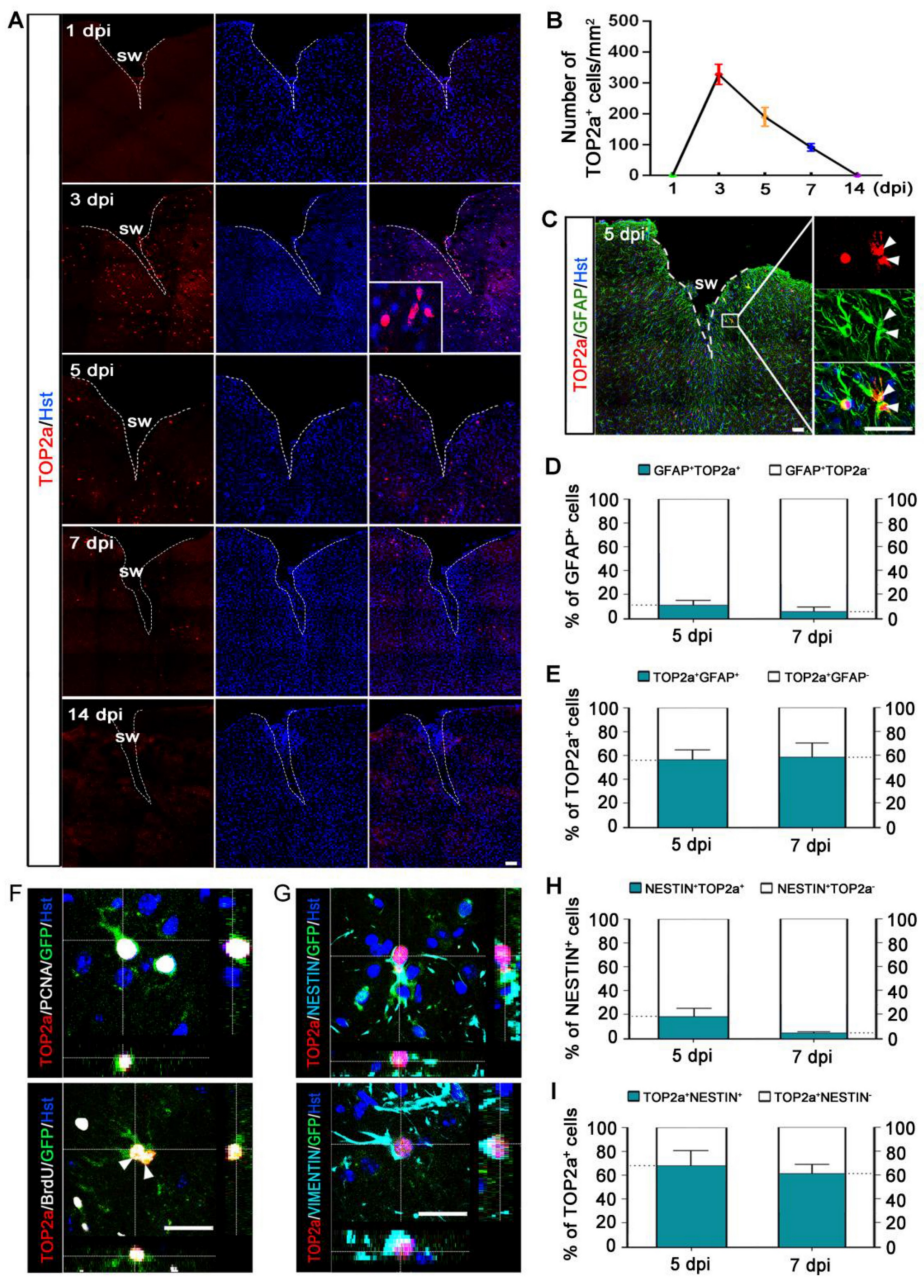
** Injury-induced expression of TOP2a in the cerebral cortex after stab wound. (A)** Time-course analysis of TOP2a expression in injured cortex at the indicated time points.** (B)** Quantification of TOP2a positive cells. At least 4 mice for each time point after injury. **(C-E)** Immunostaining showed that TOP2a was expressed in GFAP^+^ reactive astrocytes. Data from (C) was quantified in (D) and (E) (n = 4). **(F and G)** The expression of TOP2a, proliferative markers (PCNA and BrdU), and stem cell markers (VIMENTIN and NESTIN) were detected in GFP-traced reactive astrocytes in injured cortex of Gfap-Cre;Rosa-GFP mice at 5 dpi. **(H and I)** Percentage and phenotype analysis was performed by staining with TOP2a and NESTIN at 5 and 7 dpi (n = 4). White dashed lines indicate the injured area. SW, stab wound. Arrowheads in (C) and (F) indicate a pair of daughter astrocytes at late telophase, respectively. Scale bars: 50 µm.

TOP2a is found in abundance in rapidly dividing undifferentiated cells (Figure S2), and its activity is crucial for their pluripotency and differentiation potential 21, 30, 31. Our previous study showed that TOP2a is widely expressed in developing and postnatal brain and exclusively expressed in the neurogenic niches in adult brain 24. As shown in Figure S3A, immunohistochemistry revealed that TOP2b expression was broadly distributed throughout the brain of adult mouse. Unlike TOP2b, TOP2a was only expressed in the germinal niches such as SVZ, and no TOP2a signal was detected in the cerebral cortex of adult brain (Figure S3A). Strikingly, TOP2a was induced to express in the peri-lesion area of injured cortex but not in the contralateral intact cortex after stab wound (Figure S3B, Figure 3A). Time-course analysis showed that TOP2a expression peaked at 3 dpi and gradually decreased afterwards, ultimately disappearing at 14 dpi (Figure 3A and 3B).

To determine whether TOP2a was expressed in astrocytes around the injury site, immunohistochemical analysis was performed. We observed that TOP2a-positive cells expressed astroglial marker GFAP but not neuronal marker NeuN, suggesting that TOP2a was elicited to express in reactive astrocytes after stab wound (Figure 3C, Figure S4A). As shown in Figure S4B, TOP2a was firstly detected in reactive astrocytes at 3 dpi. At 5 and 7 dpi, we found that about 11% and 6% of the GFAP-positive reactive astrocytes adjacent to lesion site expressed TOP2a, respectively (Figure 3D). Of the TOP2a-expressing cells, around 56% and 59% were immunoreactive for GFAP at 5 and 7 dpi, respectively (Figure 3E, Figure S4B).

It was of note that TOP2a was co-stained with the NSC-related marker proteins, NESTIN and SOX2 (Figure S4C). Using the Gfap-Cre;Rosa-GFP tracing mice, we next examined whether stab wound-induced TOP2a was expressed in the subpopulation of reactive astrocytes that acquired stem cell properties. As shown in Figure 3F, TOP2a expression was detected in GFP-traced PCNA^+^ or BrdU^+^ reactive astrocytes. Interestingly, pairs of reactive astrocyte daughter cells could be observed in the injured cortex of both wild-type and Gfap-Cre;Rosa-GFP mice, and they were TOP2a positive, suggestive of a key role of TOP2a in the proliferation of stab wound-induced reactive astrocytes (Figure 3C and 3F). Immunohistochemical analysis also revealed that GFP-labeled reactive astrocytes were immunoreactive for both TOP2a and stem cell markers, NESTIN and VIMENTIN (Figure 3G). As shown in Figure 3H, there were about 18% and 5% of NESTIN-expressing reactive astrocytes were TOP2a-positive at 5 and 7 dpi, respectively. Of the TOP2a-positive cells, around 68% and 61% were immunoreactive for NESTIN at 5 and 7 dpi, respectively (Figure 3I). All these findings indicate that TOP2a is elicited to express in reactive astrocytes after TBI, suggestive of functional significance for their stem cell response.

### TOP2a inhibition antagonize the stem cell response in reactive astrocytes* in vitro*

**Figure 4:**
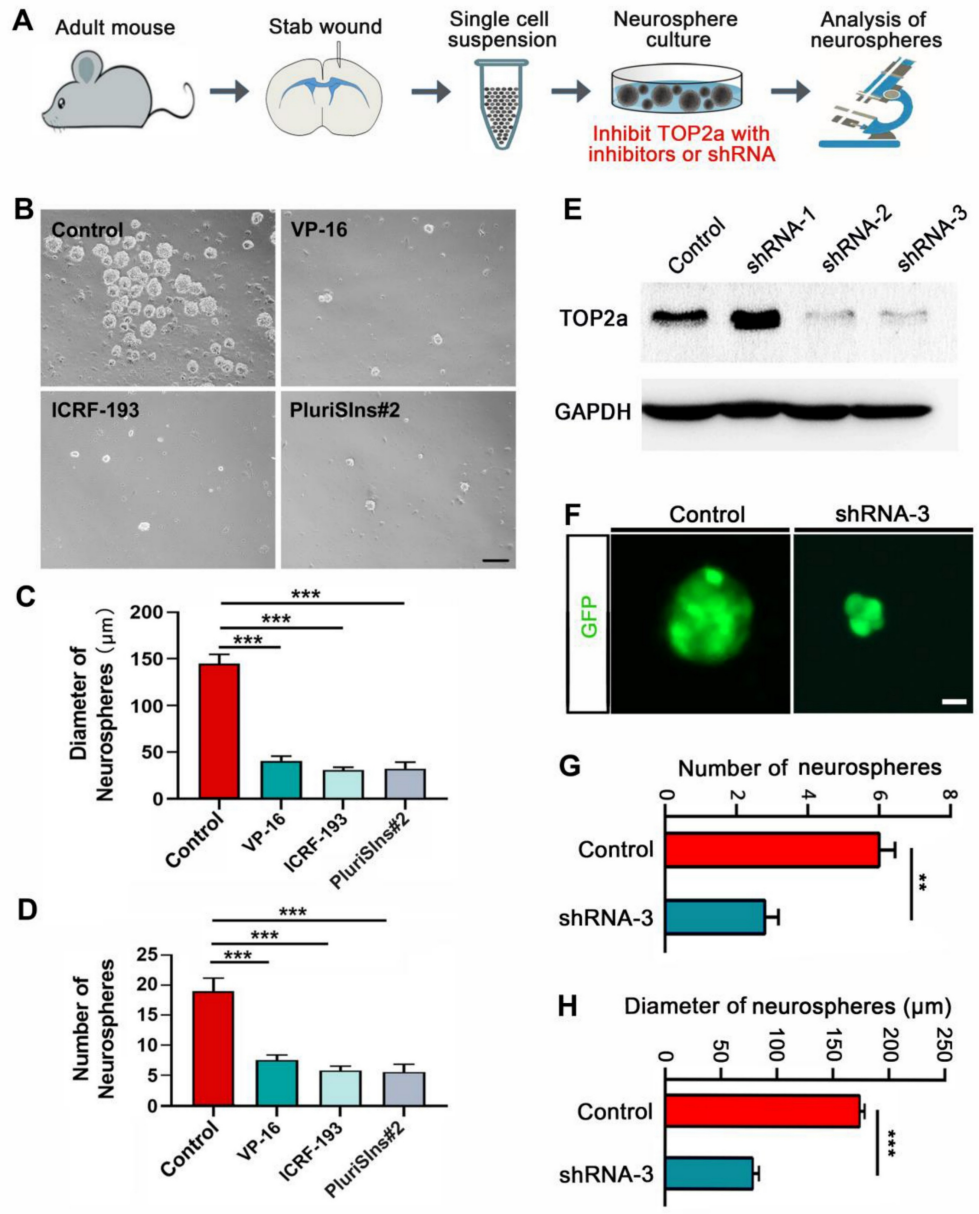
** Effects of TOP2a inhibition on the stem cell properties of reactive astrocytes *in vitro*. (A)** Experimental procedures for B-H.** (B-D)** Numbers and diameters of secondary neurospheres formed in cells isolated from stab-wounded cerebral cortex were analyzed after treated with DMSO (Control) or TOP2a inhibitors, VP-16, ICRF-193 and PluriSIns#2. **(E-H)** The shRNA-mediated knockdown of TOP2a expression impeded the formation of primary neurospheres. The knockdown efficiency of Top2a shRNA was determined by western blotting (E). Representative micrographs showed GFP-labeled neurospheres generated by injured cortex-derived cells after transduction of shRNA (F). Data from (F) were quantified in (G) and (H). **P < 0.01 and ***P < 0.01 by ANOVA with Tukey's post-hoc test or Student's *t*-test (n = 4 per condition). Scale bars: 200 µm (B) and 50 µm (F).

The stab wound-induced expression of TOP2a in reactive astrocytes prompted us to investigate whether it is necessary for acquiring the stem cell properties. TOP2a was inhibited with inhibitors or shRNA (Figure 4A). As shown in Figure 4 B-D, quantitative analysis of cultures prepared from stabbed cortex at 5 dpi showed that neurosphere formation was significantly blocked by the addition of 500 nM VP-16 or ICRF-193, the known inhibitors of TOP2 that may inhibit both TOP2a and TOP2b 21. Unlike VP-16 and ICRF-193, PluriSIns#2 is a specific inhibitor of TOP2a that selectively represses its transcription and thereby reduces TOP2a protein levels 32. Treatment of cultures with 20 μM PluriSIns#2 significantly inhibited sphere formation by cells surrounding the stab wound site (Figure 4B-D). The effect of TOP2a inhibitors on neurosphere formation was further corroborated by experiments with RNA interference. We constructed three short hairpin RNA (shRNA) against Top2a in the lentiviral vectors in which gene expression was under the control of a human GFAP promoter to specifically target reactive astrocytes. Among the three shRNAs, western blot showed that the expression of TOP2a in neurosphere-forming reactive astrocytes were dramatically knocked down by shRNA-2 and shRNA-3 (Figure 4E). We then used shRNA-3 to silence Top2a in reactive astrocytes. After cultures were transduced with shRNA-3, both the numbers and the diameters of neurospheres were significantly decreased (Figure 4F-H). Taken together, these results provide evidence that TOP2a is essential for the stem cell response in reactive astrocytes.

### Genome-wide transcriptional analysis of reactive astrocytes' stem cell response after Top2a inhibition

**Figure 5:**
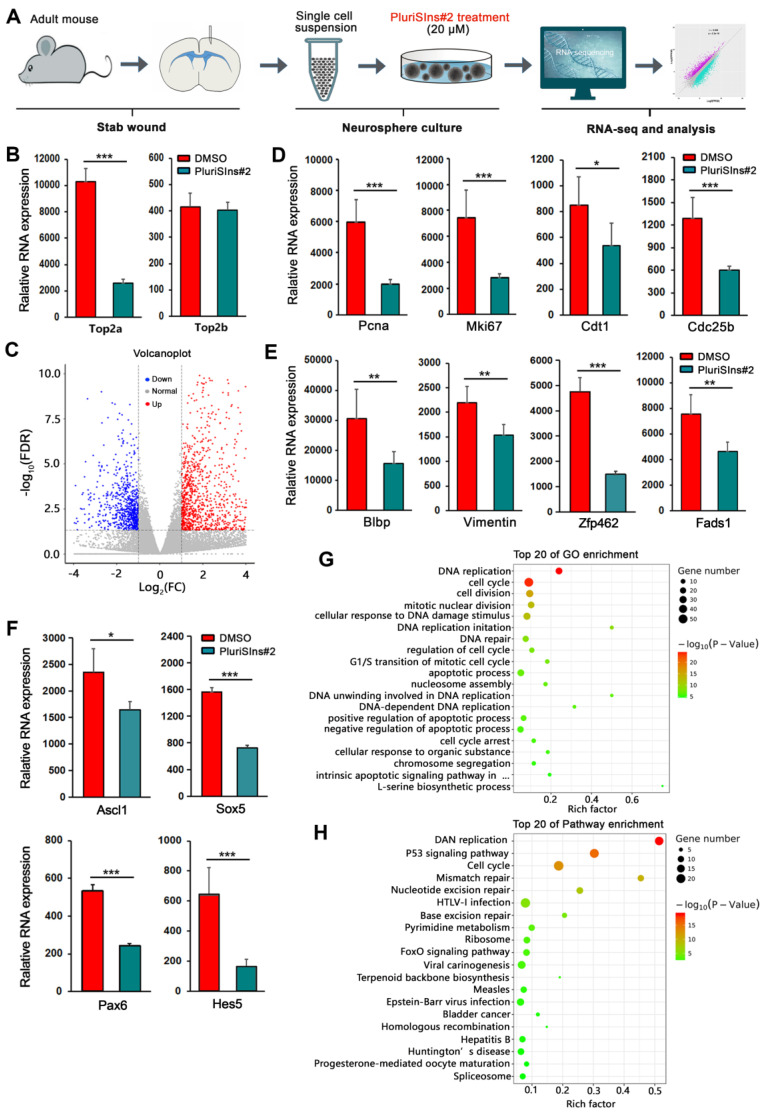
** RNA-seq analysis of TOP2a-mediated stem cell response in reactive astrocytes. (A)** Experimental procedures. **(B)** Histograms showed that the transcriptional level of Top2a but not Top2b was specifically inhibited by PluriSIns#2. **(C)** Volcano plot showed differentially expressed genes in neurospheres after treated with DMSO and PluriSIns#2. Upregulated genes were highlighted in red, while down regulation genes were highlighted in blue. **(D-F)** Quantification of gene expression indicated genes representing proliferation (Pcna, Mki67, Cdt1 and Cdc25b), multipotency (Blbp, Vimentin, Zfp462 and Fads1) as well as transcription regulation (Ascl1, Sox5, Pax6 and Hes5) were downregulated after PluriSIns#2 treatment. **(G and H)** GO and pathway enrichment analysis showed the significant changes of gene function and pathways between the two groups, respectively. *P < 0.05, **P < 0.01 and ***P < 0.001 by Student's *t*-test (n = 4 per condition).

To further globally understand the role of TOP2a in reactive astrocytes' stem cell response, we performed RNA Sequencing to analyze the genome-wide transcriptional profiling of TBI-induced reactive astrocytes-derived neurospheres after down-regulation of TOP2a with PluriSIns#2 (Figure 5A). Three days after cultured neurospheres were treated with PluriSIns#2 or DMSO, cells were collected for bulk transcriptomic RNA-Seq. As shown in Figure 5B, we found that PluriSIn#2 significantly reduced RNA expression of Top2a but not Top2b in neurospheres, further indicating its ability to downregulate Top2a exclusively. Compared with the DMSO group, RNA-Seq analysis showed that there was a substantial difference in PluriSIns#2-treated neurospheres, including 715 up-regulated genes and 353 down-regulated genes (Figure 5C and Figure S5).

Self-renewal and multipotency are the two main characteristics of NSCs. Figure 5D revealed that PluriSIns#2-mediated inhibition of TOP2a resulted in a significant decrease in the transcriptional level of cell proliferation-related genes including Pcna, Mki67, Cdt1 and Cdc25b. Of note, PluriSIns#2 treatment also dramatically shut down the gene expression of multipotential markers (Blbp, Vimentin, Zfp462 and Fads1) as well as transcription factors (Ascl1, Sox5, Pax6 and Hes5) (Figure 5E and 5F). GO analysis on the DEGs indicated that they were primarily involved in the biological process of cell proliferation, such as DNA replication, cell cycle, cell division, mitotic nuclear division, DNA replication imitation, and regulation of cell cycle (Figure 5G). KEGG analysis is a powerful tool to offer crucial insights into the regulatory signaling mechanisms that govern biological processes. As shown in Figure 5H, DNA replication, P53 and cell cycle are the main signaling pathways involved in TOP2a-mediated reactive astrocytes' stem cell response. All these findings provide molecular evidence for the essential roles of Top2a in the self-renewal and multipotency of reactive astrocyte-derived neurospheres and that Top2a downregulation is deleterious to the maintenance of their stemness properties.

### Top2a deletion disrupts the stem cell response in reactive astrocytes *in vitro*

**Figure 6:**
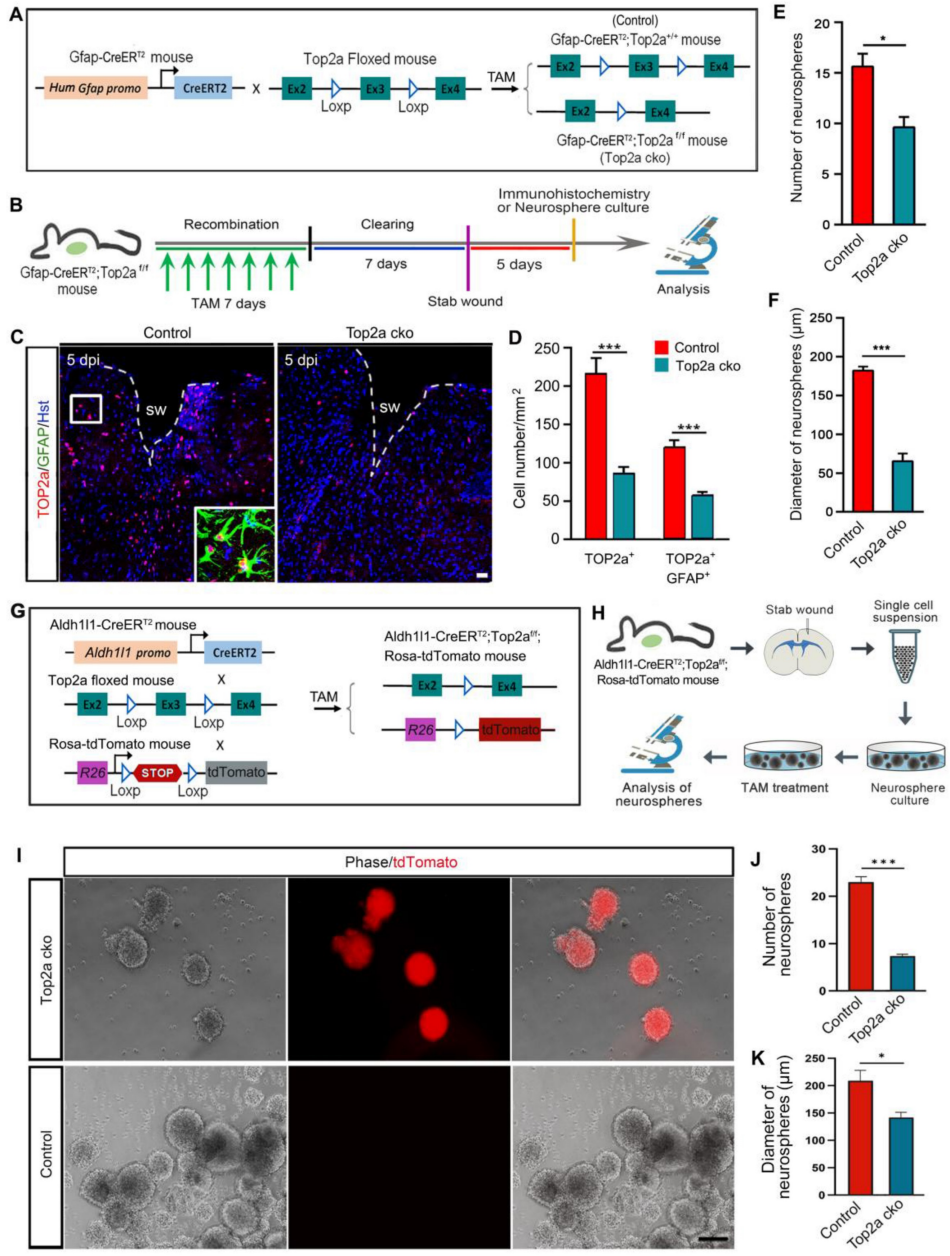
** Effects of Top2a deletion on the stem cell properties of reactive astrocytes *in vitro*. (A)** The genetic strategy to conditionally knock out Top2a gene with Gfap-CreER^T2^ in astrocytes. The littermates with functional floxed allele were used as a control (Gfap-CreER^T2^;Top2a^+/+^) for the Top2a cko (Gfap-CreER^T2^;Top2a^f/f^) mice with inactivated allele. TAM, tomaxifen. **(B)** Schematic diagrams of experimental procedures for (C-F).** (C and D)** Expression of TOP2a in GFAP^+^ reactive astrocyte was significantly knocked down in the Top2a cko mice with stab wound, compared with that in control mice. **(E and F)** Quantitative analysis of the numbers and diameters of neurospheres showed that conditional deletion of Top2a inhibited the formation of neurospheres. Cells were isolated from stab-wounded cerebral cortex of Gfap-CreER^T2^;Top2a^f/f^ (Top2a cko) or Gfap-CreER^T2^;Top2a^+/+^ (control) mice. **(G)** The genetic strategy to conditionally knock out Top2a gene with Aldh1l1-CreER^T2^ in astrocytes.** (H)** Schematic diagrams of experimental procedures for (I-K). Cells were isolated from stab-wounded cerebral cortex of Aldh1l1-CreER^T2^;Top2a^f/f^;Rosa-tdTomato mice and treated with TAM (Top2a cko) or ethanol (control). **(I-K)** Quantitative analysis of the numbers and diameters of neurospheres. *P < 0.05 and ***P < 0.001 by Student's *t*-test (n = 4 per condition). Scale bars: 50 µm (C) and 200 µm (I).

In addition to inhibitor- and shRNA-mediated inhibition of TOP2a *in vitro*, we generated an inducible knockout mouse to further examine whether removal of Top2a from reactive astrocytes affected their capacity to form neurospheres. The Gfap-CreER^T2^ mouse was crossed with Top2a floxed mouse to conditionally delete Top2a gene in reactive astrocytes by treatment with TAM (Figure 6A). The littermates with functional floxed allele were used as a control (Gfap-CreER^T2^;Top2a^+/+^) for the Top2a cko (Gfap-CreER^T2^;Top2a^f/f^) mice with inactivated allele. After seven-day tomaxifen clearing, animals were subjected to stab injury (Figure 6B). Compared with that in control mice, histochemical analysis showed that the number of TOP2a^+^ or TOP2a^+^/GFAP^+^ cells surrounding lesion site was significantly decreased in Top2a cko mice at 5 dpi, suggesting that Top2a was specifically and effectively knocked out in astrocytes (Figure 6C and 6D). We isolated reactive astrocytes surrounding the stab wound at 5 dpi, and then probed their potential for neurosphere formation (Figure 6B). Quantitative assessment of cultures revealed a significant reduction in the number of neurospheres formed by cells from Top2a cko mice compared with that from control animals (Figure 6E). Notably, Top2a cko also resulted in a drastic decrease in the diameter of neurospheres (Figure 6F).

In order to trace reactive astrocytes while selective knocking out Top2a, we generated another transgenic mouse line. As shown in Figure 6G, the Aldh1l1-CreER^T2^ mouse and the fluorescent reporter line (Ai14, Rosa-tdTomato mouse) were crossed with the floxed Top2a alleles (Top2a floxed mouse) to obtain the Aldh1l1-CreER^T2^;Top2a^f/f^;Rosa-tdTomato transgenic mouse. Five days after stab wounding, reactive astrocytes were isolated from stabbed cortex of the Aldh1l1-CreER^T2^;Top2a^f/f^;Rosa-tdTomato transgenic mice and applied to neurosphere culture (Figure 6H). The cultures were treated with 1 µM TAM to induce recombination in the CreER^T2^/loxP system and selectively delete Top2a (Top2a cko), while the cultures treated with ethanol were used as control (Figure 6H). Figure 6I-K showed that both the numbers and the diameters of neurospheres were significantly decreased in Top2a cko group. Collectively, these data obtained from Gfap-CreER^T2^;Top2a^f/f^ and Aldh1l1-CreER^T2^;Top2a^f/f^;Rosa-tdTomato transgenic mice suggest a direct effect of TOP2a on the stem cell potential of stab wound-induced reactive astrocytes *in vitro*.

### TOP2a is essential for the stem cell response in reactive astrocytes* in vivo*

**Figure 7:**
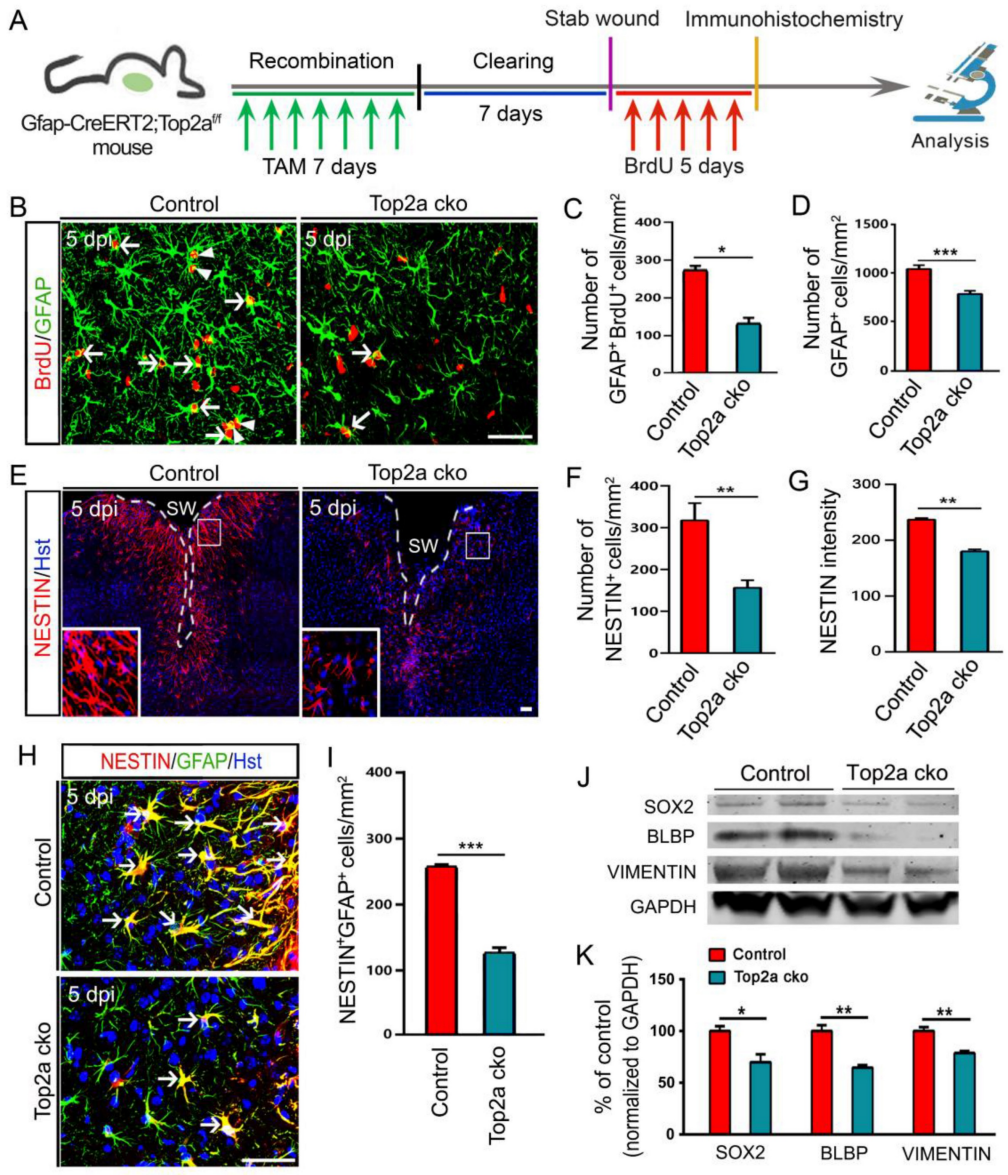
** Effects of TOP2a deletion on the stem cell response in reactive astrocytes in injured cortex. (A)** Schematic diagrams of experimental procedures. The littermates with functional floxed allele were used as a control (Gfap-CreER^T2^;Top2a^+/+^) for the Top2a cko (Gfap-CreER^T2^;Top2a^f/f^) mice with inactivated allele.** (B-I)** Immunohistochemical analysis showed that the proliferation and stem cell response of reactive astrocytes was significantly inhibited by Top2a cko in injured cortex. The histograms in (C and D), (F and G) and (I) provide a quantitative summary of the data per area in a section for more than ten animals. **(J and K)** Western blot analysis of the expression of the stem cell markers, SOX2, BLBP, and VIMENTIN in the injured cortex of control or Top2a cko mice, isolated at 5 dpi. The histogram in (K) depicts the quantitative analysis of western blot by densitometry. *P < 0.05, **P < 0.01, and ***P < 0.001 by Student's *t*-test. Arrowheads in (B) indicate a pair of daughter astrocytes at late telophase. White dashed lines in (E) indicate the injured area. SW, stab wound. Scale bars: 50 µm.

Because of the pronounced inhibition of neurosphere formation by Top2a cko, we next asked whether removal of Top2a affected the proliferative response of reactive astrocytes *in vivo*. A shown in Figure 7A, the Gfap-CreER^T2^;Top2a^f/f^ transgenic mice were treated with TAM to conditionally delete Top2a gene in reactive astrocytes. After seven-day TAM clearing, animals subjected to stab injury received daily intraperitoneal injection of BrdU for consecutive 5 days and were applied to immunohistochemical analysis (Figure 7A). Figure 7B and 7C revealed that the number of GFAP^+^/BrdU^+^ cells surrounding the stab wound was significantly reduced in Top2a cko mice relative to control mice. Accordingly, Top2a deletion also resulted in a significant decrease in the number of GFAP^+^ reactive astrocytes (Figure 7D). Hence, these data suggest that TOP2a is necessary for the stab wound-induced proliferative reaction of reactive astrocytes *in vivo*.

We further investigated the effects of Top2a deletion on the stem cell response of reactive astrocytes in the stabbed cerebral cortex. Immunostaining showed that fewer NESTIN^+^ cells were induced around the injury site in Top2a cko mice, compared with that in control mice (Figure 7E and 7F). In the meantime, the expression level of NESTIN was significantly decreased by removal of Top2a (Figure 7G). We also counted the NESTIN^+^GFAP^+^ cells in the cortical peri-injury area. As expected, Top2a deletion resulted in a dramatic decrease in the number of NESTIN^+^GFAP^+^ cells relative to control (Figure 7H and 7I). Following stab wounding, in addition, a defined volume of injured cortex of Top2a cko or control mice was isolated to perform western blot analysis at 5 dpi. Figure 7J and 7K revealed that the expression of NSC-related marker proteins, including SOX2, BLBP and VIMENTIN, were significantly down-regulated in Top2a cko mice, compared with that in control mice. Together, these data indicate that the stab wound-induced *in vivo* stem cell reaction of reactive astrocytes was TOP2a-dependent.

### SHH signaling upregulates TOP2a to induce the stem cell response in reactive astrocytes

**Figure 8:**
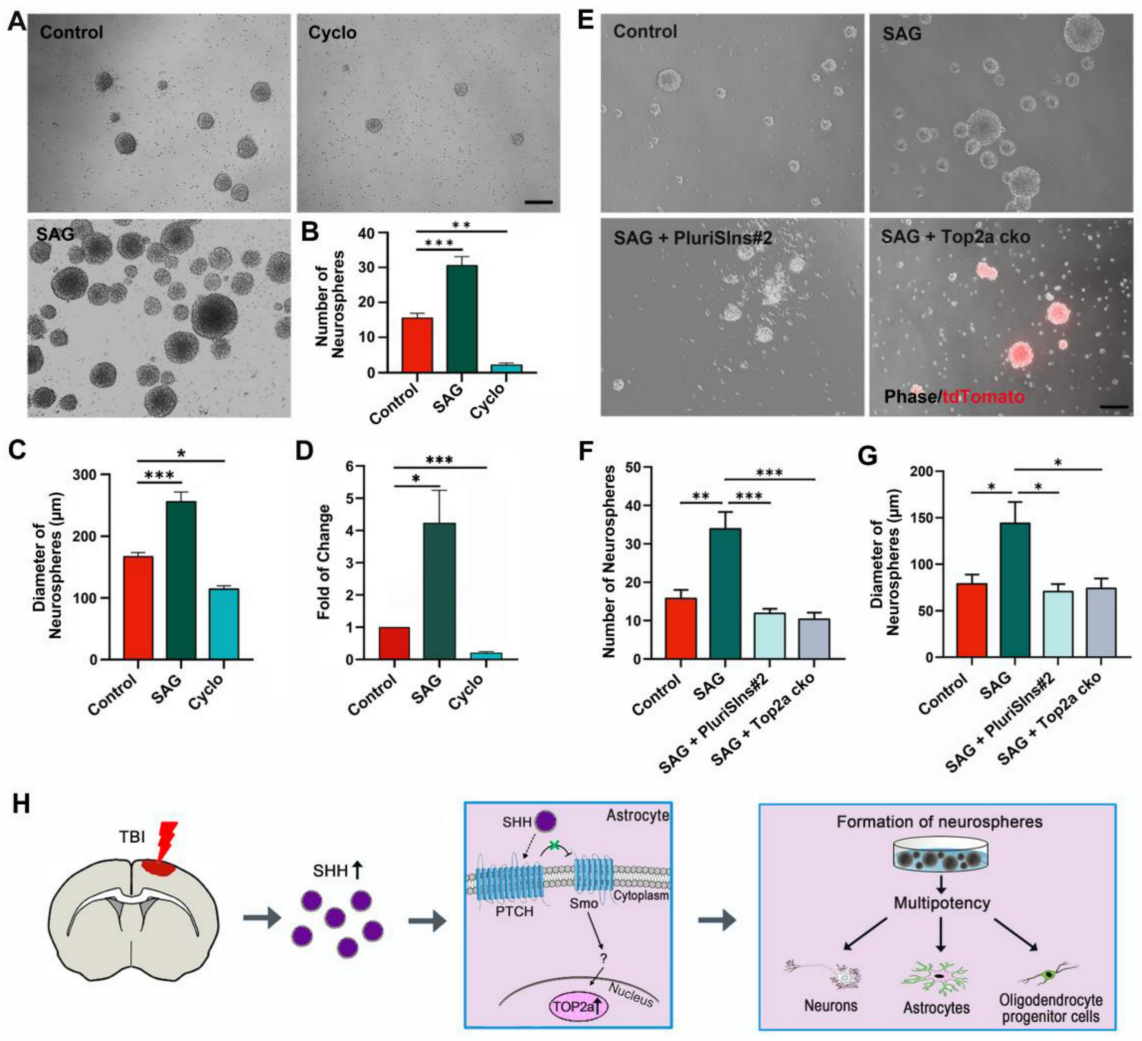
** TOP2a is indispensable for SHH signaling-mediated effects on the stem cell response in reactive astrocytes. (A-C)** Numbers and diameters of secondary neurospheres formed in cells from stab-wounded cerebral cortex were analyzed after treated with DMSO (Control), SAG and cyclopamine (Cyclo). **(D)** The levels of Top2a mRNA expression in reactive astrocytes derived from stab-wounded cerebral cortex were determined by qRT-PCR. Cells were treated with DMSO (Control), SAG and Cyclo.** (E-G)** Numbers and diameters of secondary neurospheres formed in cells from stab-wounded cerebral cortex were analyzed after treated with DMSO (Control), SAG, SAG/PluriSIns#2 and SAG/Top2a cko. **(H)** Proposed model for the role of SHH/Top2a in the stem cell response in reactive astrocytes after TBI. *P < 0.05, **P < 0.01, and ***P < 0.001 by ANOVA with Tukey's post-hoc test. Scale bars: 200 µm.

In previous studies, sonic hedgehog (SHH) was found to upregulate specifically in the injured cerebral cortex after TBI and functioned as an extracellular signal to trigger the stem cell response in reactive astrocytes 15, 20. We isolated reactive astrocytes surrounding the stab wound site at 5 dpi and treated them with 0.5 µM SAG, an activator for the SHH signal transducer Smoothened (Smo) 33. Quantitative assessment showed a significant increase in neurosphere formation in the presence of SAG in cultures compared with control cultures (Figure 8A-C). In contrast, the neurosphere formation was obviously reduced by supplementation of cultures with 5 µM cyclopamine (Figure 8A-C), a known inhibitor of SHH signaling 20. Interestingly, real-time qRT-PCR of reactive astrocytes isolated from stab-wounded cerebral cortex at 5 dpi revealed that SAG-mediated activation of SHH signaling significantly increased the level of Top2a mRNA expression, while cyclopamine-mediated inhibition of SHH signaling dramatically decreased the level of Top2a mRNA expression (Figure 8D). These findings prompted us to investigate whether the intracellular molecule TOP2a was involved in SHH signaling-mediated stem cell response.

In order to determine the functional relationship between TOP2a and SHH signaling during the stem cell response, we next asked whether inhibition of TOP2a in the *in vitro* neurosphere assay affected the SHH signaling-capacity of isolated cells to generate neurospheres. Five days after stab wound, reactive astrocytes surrounding the lesion site were isolated for the *in vitro* neurosphere assay. When SHH signaling in reactive astrocytes was activated by SAG, both the numbers and the diameters of neurospheres were significantly increased (Figure 8E-G). However, this effect could be blocked by addition of the TOP2a inhibitor PluriSIns#2 (Figure 8E-G). In addition, reactive astrocytes surrounding the lesion site were also isolated from the stab-wounded cerebral cortex of Aldh1l1-CreER^T2^;Top2a^f/f^;Rosa-tdTomato mice and used for the *in vitro* neurosphere assay. The cultures were treated with TAM to induce selective deletion of Top2a in reactive astrocytes. As shown in Figure 8E-G, we found that Top2a cko could also block this effect of SAG on the reactive astrocytes' capacity to generate neurospheres. Combining our findings of the role of TOP2a in the stem cell response in reactive astrocytes with the data about SHH signaling already available in the literatures enables us to propose a model of the molecular basis for this response (Figure 8H). After TBI, the invasive injury results in SHH upregulation in the brain. The SHH binds to the receptor complex of Patched (PTCH)-mediated Smo to initiate the downstream signaling cascade, which further elicits the expression of TOP2a and induces the stem cell potential of reactive astrocytes.

## Discussion

Increasing evidence show that parenchymal astrocytes retain a higher level of plasticity than that previously realized, especially when it comes to reactive astrocytes. For example, injury-induced reactive astrocytes have been shown to partially dedifferentiate and acquire stem cell potential, which may be considered dormant NSCs outside the neurogenic niches in adult brain 15-18. A systematic and in-depth investigation of astroglial plasticity is crucial to broaden our concept of mature astrocytes in the adult mammalian brain. In this study, we provide evidence that TOP2a is elicited to express in reactive astrocytes after TBI and functions as a key intrinsic regulatory factor for the astroglial proliferative and stem cell responses to injury.

Astrocyte-like cells (radial glia) acting as neural stem cells in adult neurogenic niches, such as SVZ and SGZ, have been intensively studied in both normal and injured brains 5, 6, 34. However, the stem cell response of injury-induced reactive astrocytes outside the neurogenic niches, in particular the underlying mechanism, is only beginning to be appreciated. Following diverse type of CNS injury, astrocytes are activated and become reactive 18, 35. Interestingly, subpopulation of these reactive astrocytes is shown to acquire stem cell properties upon acute invasive brain injury, such as trauma or stroke 14, 15, 27, 36-38. Consistent with previous studies, our data demonstrated here that stab wound injury resulted in reactive astrogliosis and converted mature astrocytes into an immature state. Although quiescent mature astrocytes are postmitotic in adult brain, some reactive astrocytes were observed to resume proliferation and share some characteristics with NSCs and developmental radial glia, such as expression of NESTIN, SOX2, BLBP and VIMENTIN. Importantly, the stab wound-derived cortical reactive astrocytes were able to form self-renewing and multipotent neurospheres *in vitro*. These findings thereby support the idea that mature astrocytes can be dedifferentiated toward earlier developmental stages, such as in the postnatal or embryonic brain. It should be noted that reactive astrocytes were phenotypically heterogeneous in the injured brain and only a fraction of them dedifferentiated into NSC-like cells. For instance, some reactive astrocytes resumed proliferation upon stab wound injury, but many of them did not. We observed that there were only about 54% and 28% GFAP-positive reactive astrocytes expressing NSC marker NESTIN in the stabbed cerebral cortex at 5 and 7 dpi, respectively. The fate mapping also showed that not all GFP-traced reactive astrocytes expressed NSC-related proteins, NESTIN, BLBP, and VIMENTIN. In future studies, therefore, it is important to identify the specific subset of reactive astrocytes that undergo a functional dedifferentiation and acquire stem cell potential in response to brain injury.

Although reactive astrocytes were shown to dedifferentiate into an NSC-like immature state in response to TBI, the underlying molecular mechanism remains elusive. The failure of astrocytes to display proliferative and stem cell reaction in the contralateral side of the cortex after stab wound prompted us to identify key factors or signals in the injured cortex that control the dedifferentiation of reactive astrocytes. Of note, the high levels of SHH known to enter the brain from extraneural sources after invasive injury has been reported to trigger this stem cell response of reactive astrocytes 15. However, it is unknown whether there is any intrinsic regulatory factor of the acquisition of stem cell potential in reactive astrocytes. As one of the most conserved proteins, TOP2a has been traditionally reported to play key roles in relieving torsional stress of DNA during fundamental biological process 19, 39-41. However, its function in gene regulation, especially in the context of maintaining stem cell properties, is just beginning to be understood. By chromatin immunoprecipitation for TOP2a in mouse embryonic stem cells (mESCs), Thakurela et al. showed that the targets of TOP2a are mainly involved in proliferation and pluripotency, and TOP2a activity is crucial for pluripotency and differentiation potential of mESCs 21. Recently, we found that TOP2a was absent in cerebral cortex and exclusively present in endogenous stem cell niches in adult brain 24. In this study, TOP2a was elicited to express in reactive astrocytes after stab wound injury. All these findings provided a hint that TOP2a might play essential roles in the astroglial proliferative and stem cell responses to brain injury. Notably, the potential of reactive astrocytes to form neurospheres *in vitro* was dramatically impaired by inhibiting TOP2a with pharmacological inhibitors or shRNA. Importantly, conditional knock-out mouse studies showed selective deletion of Top2a in reactive astrocytes not only inhibited their neurosphere-forming capacity *in vitro*, but also inhibited their proliferative and stem cell response *in vivo*. Taken together, our study identifies TOP2a as a key intrinsic regulatory factor of the acquisition of stem cell potential in reactive astrocytes. Importantly, we also provided evidence that TOP2a might serve as a downstream factor of SHH signaling to induce the stemness response in reactive astrocytes, although further refinement of the relationship between TOP2a and SHH signaling is required.

The subpopulation of injury-induced reactive astrocytes that resume proliferation and take on an NSC character has been considered as dormant NSCs outside the adult neurogenic niches and has gained an unprecedented attention for their potential application in endogenous brain cell replacement upon damage. However, the functional similarities and differences between reactive astrocytes-derived NSC-like cells and “true” NSCs in neurogenic niches remain to be better understood. It is noteworthy that reactive astrocytes-derived NSC-like cells are restricted from neuronal differentiation *in vivo*, despite their multipotency and capacity for self-renewal in culture, suggesting that they may not be bona fide NSCs 14, 27. Indeed, reactive astrocytes are shown to re-express some genes related to neurogenesis, such as CD24, Sox4, Sox11 and Ptbp2 17. Compared with endogenous embryonic or adult NSCs, however, these genes are expressed at much lower levels in reactive astrocytes 17. In our study, TOP2a was elicited to express in reactive astrocytes and instructed them to acquire NSC-related potential, while TOP2a expression ultimately disappeared at 14 dpi. Therefore, the brain injury-induced conversion of mature astrocytes into an immature state might be a transient feature, which is just an intermediate process of reactive gliosis. Given their similarities with stem cells, however, the highly plastic and immature set of reactive astrocytes may present potential targets for neuronal reprogramming *in situ* after brain injury. The injury-induced reactivation is like to involve epigenetic modifications, in which TOP2a re-expressed in reactive astrocytes may keep the chromatin in an accessible state, thereby more susceptible to the activity of neurogenic genes 18, 21. In fact, forced expression of transcription factor NEUROG2 or DLX2 in astroglia-derived neurosphere cells obtained from lesioned cortex can induce many of them reprogramming into glutamatergic and GABAergic neurons, respectively 42. In addition, the lineage restriction for reactive astrocytes-derived NSC-like cells may be also due to the local environment. Recently, we observed that the expression level of NOTCH1 in spinal cord was dramatically upregulated after injury, peaking at 7 dpi. Intriguingly, blocking NOTCH1 could successfully convert reactive astrocytes into neuronal cells 24. Therefore, future studies are necessary to unravel the intracellular and extracellular cues that control the lineage restriction for reactive astrocytes-derived NSC-like cells *in vivo*.

In summary, we here identified TOP2a as a key intrinsic regulator of the acquisition of stem cell potential in reactive astrocytes, enhancing our understanding of the molecular process underlying the reactivation and dedifferentiation of local astroglia at lesion site with the final aim of harnessing reactive astrocyte for endogenous CNS repair after injury.

## Supplementary Material

Supplementary figures and tables.
