# Supplementary material for: Topoisomerase IIα-mediated stemness response in reactive astrocytes to traumatic brain injury
Source: Theranostics. 2025 Apr 13;15(11):5402–19. doi: 10.7150/thno.111923 (PMC12036888; doi:10.7150/thno.111923)
Supplement: Supplementary file 1 — Supplementary figures and tables. [file thnov15p5402s1.pdf]

Supplementary Information

Topoisomerase IIα-mediated stemness response in reactive astrocytes to traumatic brain injury

Shangyao Qin, Xiao Huang, Yimin Yuan, Hong Liu, Jiali Li, Ziwei Dai, Zhida Lan, Yingyan Pu, Cheng He, Zhida Su

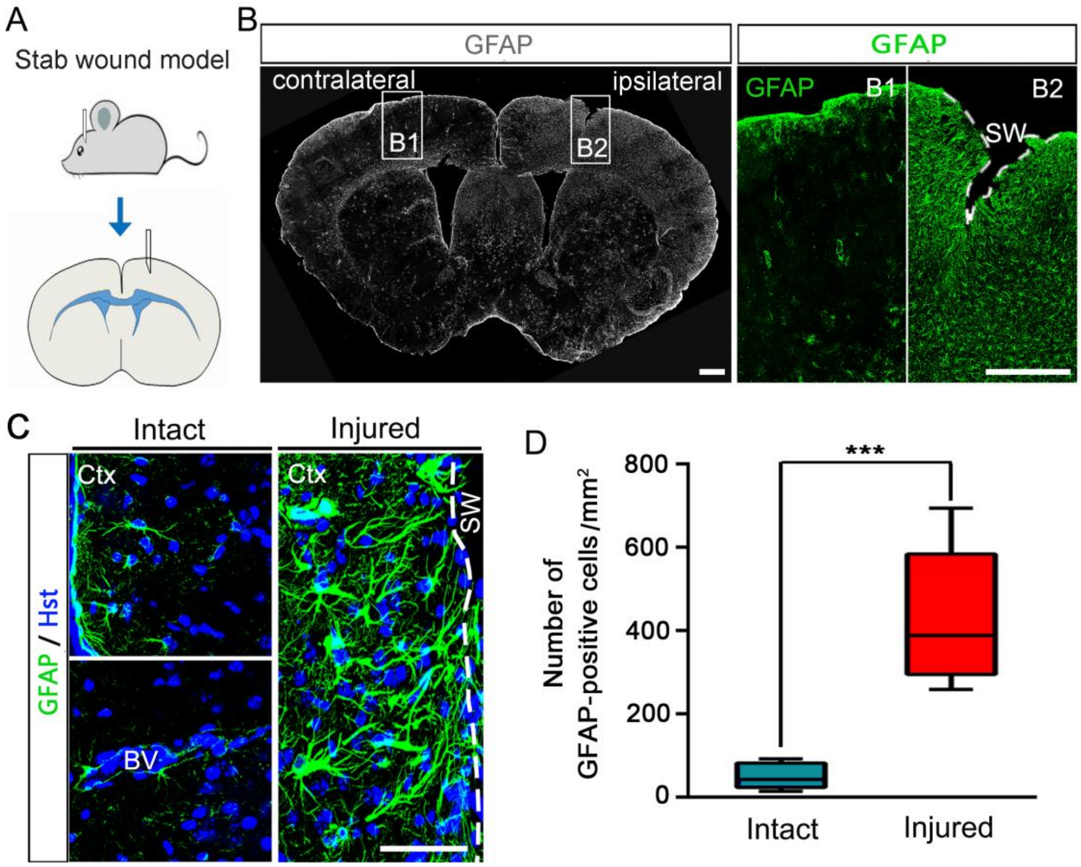

Figure S1. Reactive response of astrocytes to stab wounding in the cerebral cortex. (A) Schematic diagram of stab wound injury performed in the cerebral cortex. **(B and C)** Immunohistochemical analysis of astroglial response in the cortical region contralateral (intact) or ipsilateral (injured) to a stab wound. **(D)** Quantification of GFAP-positive cells per area in intact and injured cerebral cortex. ***P < 0.001 by Student's *t*-test (intact group, n = 9; injured group, n = 12). White dashed lines indicate the injured area. SW, stab wound; Ctx, cortex; BV, blood vessel. Scale bars: (B), 300 µm; (C), 50 µm.

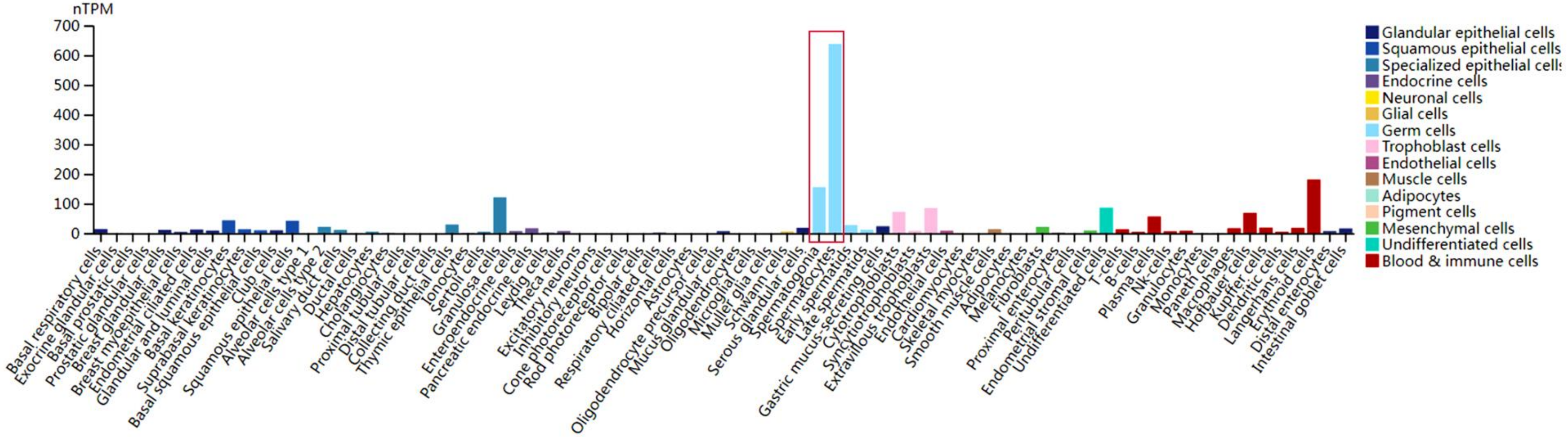

Figure S2. Top2a RNA expression in single cell types. The histogram showed a summary of normalized single cell RNA (nTPM) from all single cell types. Color-coding is based on cell type groups, each consisting of cell types with functional features in common. Red box indicates the cell type with high expression level of Top2a. Data were obtained from Human Protein Atlas Dataset available from https://www.proteinatlas.org.

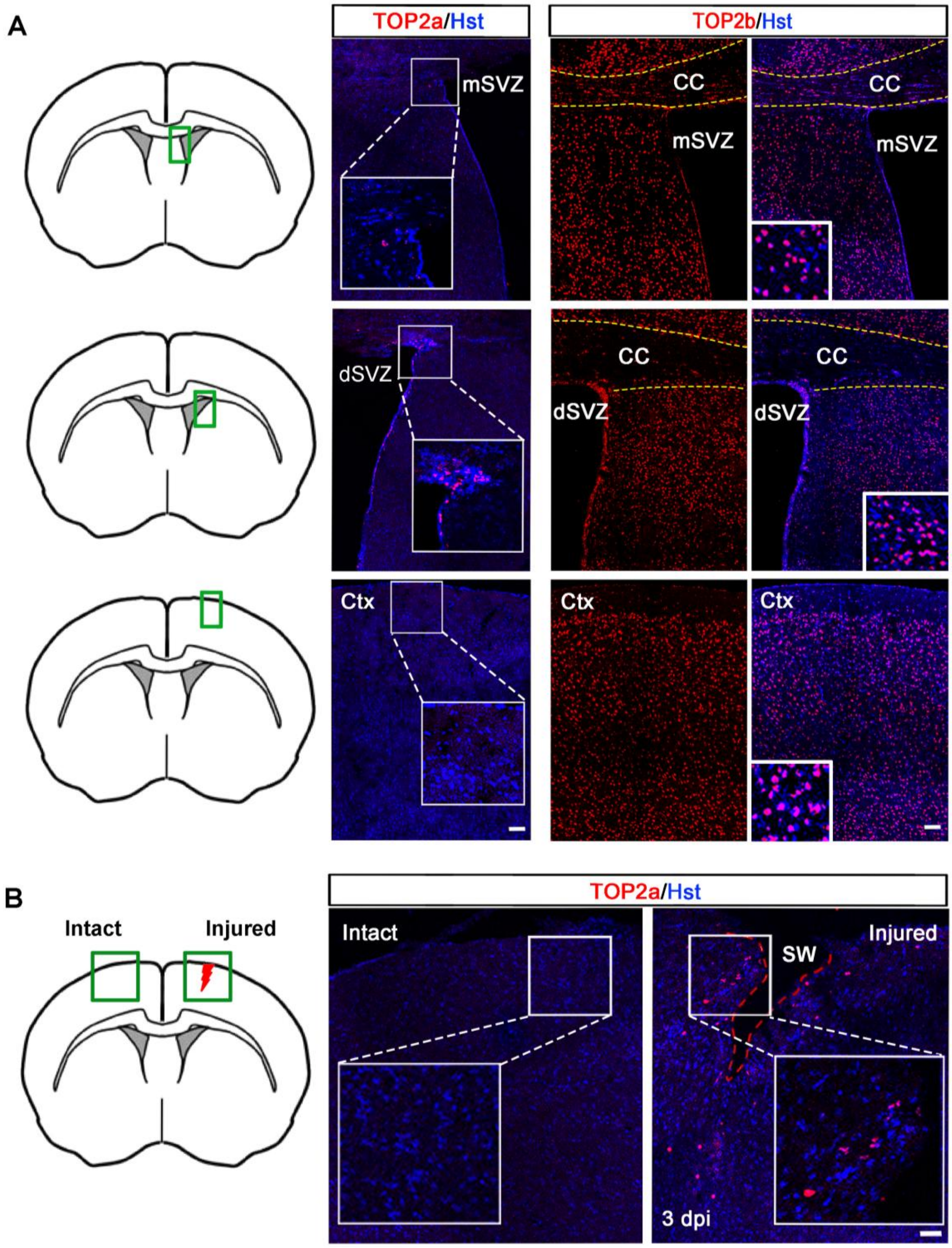

Figure S3. Expression of TOP2a and TOP2b in adult brain. (A) Immunohistochemical analysis showed that TOP2a expression was limited to the neurogenic regions, while TOP2b was broadly expressed in brain. mSVZ, medial subventrical zone; dSVZ, dorsal subventrical zone; Ctx, cortex; CC, corpus callosum. **(B)** TOP2a expression was induced in the injured cortex after stab wound. Yellow and red dashed lines indicate the corpus callosum and the injured area, respectively. SW, stab wound. Scale bars: 50 μm.

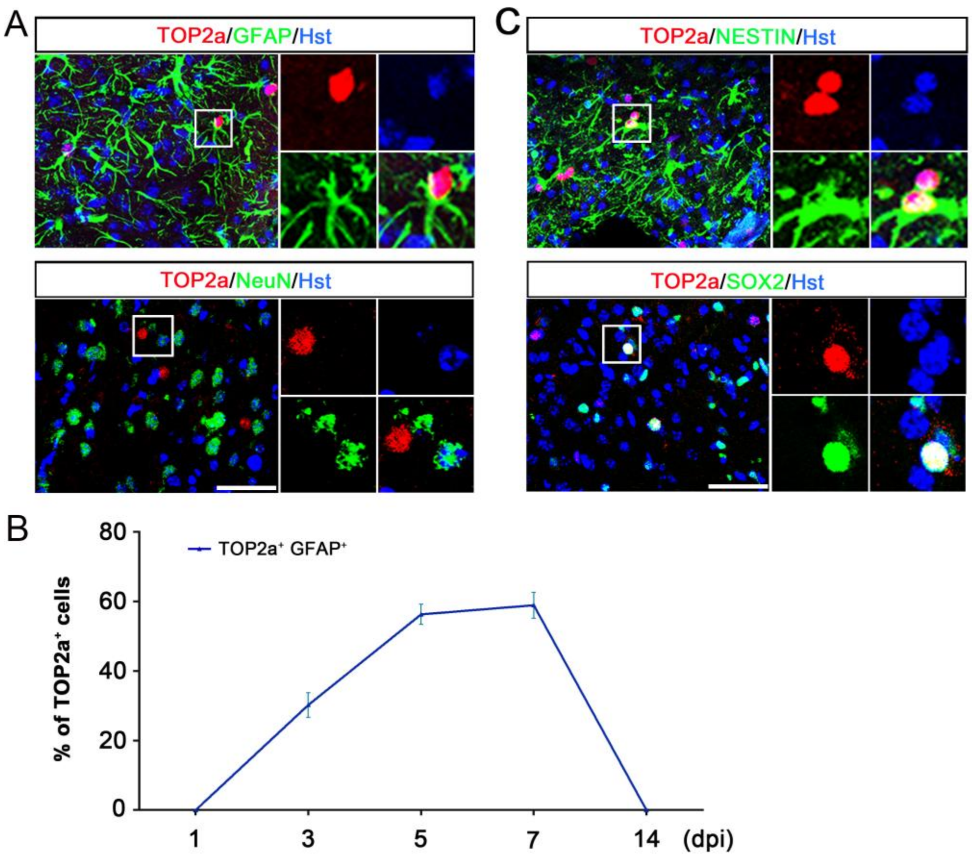

Figure S4. Expression of TOP2a in neural cells. (A and B) Immunohistochemical analysis of TOP2a expression in astrocytes (GFAP$^+$) and neurons (NeuN$^+$). Data from (A) were quantified in (B) (n = 3 for each time point). **(C)** Immunostaining of TOP2a expression in NESTIN$^+$ or SOX2$^+$ cells. Scale bars: 50 μm.

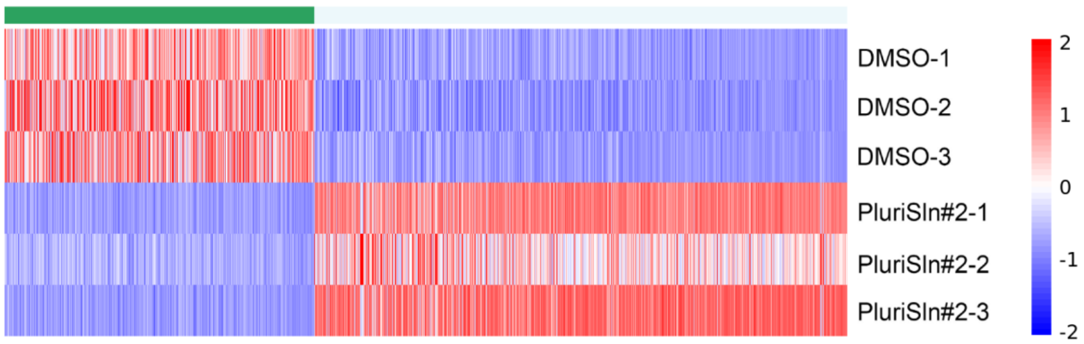

Figure S5. RNA-Seq analysis of the transcriptional changes in neurospheres after TOP2a inhibition with PluriSIns#2.

Table S1. Primary antibodies used for immunostaining

Antibody	Host	Dilution	Source	Catalog number
ALDH1L1 (Aldehyde dehydrogenase 1 L 1)	Mouse	1:100	Abcam	Ab56777
BLBP (Brain lipid binding protein)	Rabbit	1:100	Abcam	Ab32423
BrdU (5-bromodeoxyuridine)	Rat	1:100	Abcam	Ab6326
GFAP (Glial fibrillary acidic protein)	Mouse	1:200	Sigma	G3893
GFP (Green fluorescent protein)	Chicken	1:200	Aves Labs	1020
IBA1 (Ionized calcium binding adapter molecule 1)	Rabbit	1:100	Wako	019-19741
NeuN (Neuron-specific nuclear protein)	Mouse	1:100	Millipore	MAB377
NESTIN	Rat	1:100	Santa Cruz	sc-33677
NG2 (New glue 2)	Rabbit	1:200	Millipore	AB5320
PCNA (Proliferating cell nuclear antigen)	Rat	1:1000	Chromotek	16D10
SOX2 (SRY-box containing gene 2)	Goat	1:100	R&D	AF2018-SP
SOX10 (SRY-box containing gene 10)	Rabbit	1:100	Cell Signaling Technology	78330
TOP2a (Topoisomerase II alpha)	Rabbit	1:100	Abcam	Ab52934
TOP2b (Topoisomerase II beta)	Mouse	1:200	Abcam	Ab235898
TUBB3 (Tubulin beta-3)	Rabbit	1:200	Abcam	Ab18207
VIMENTIN	Mouse	1:100	Abcam	Ab8978

Table S2. Primary antibodies used for western blot

Antibody	Host	Dilution	Source	Catalog number
BLBP (Brain lipid binding protein)	Rabbit	1:1000	Abcam	ab32423
GAPDH (Glyceraldehyde phosphate dehydrogenase)	Rabbit	1:2000	Abcam	ab9485
GFAP (Glial fibrillary acidic protein)	Goat	1:5000	Abcam	ab53554
SOX2 (SRY-box containing gene 2)	Goat	1:200	R&D	AF2018-SP
TOP2a (Topoisomerase II alpha)	Rabbit	1:1000	Abcam	ab52934
VIMENTIN	Mouse	1:200	Abcam	ab8978
